# Non-Panel Men and the "Medical Directory"

**Published:** 1914-05-09

**Authors:** Arthur J. Brock

**Affiliations:** Edinburgh


### THE EDITOR'S LETTER-BOX.

### Non-Panel Men and the "Medical Directory."

*To the Editor of* THE HOSPITAL.

SIR,—I gladly accept Dr. F. L. Nicholls's statement that, when he said the non-panel men were "entitled to some distinction," he did not mean that the fact of their not being on the panel rendered them superior to the panel men as such. One must make all allowance for the natural irritation many men feel at seeing their patients going "over the way" to a neighbour who has resiled from his pledged word.

Doubtless the *débâcle* was started, in the first instance, by men with essentially mercenary, or, at least, political motives, but, when it was once under way, hundreds were quite willy-nilly drawn into the vortex—not merely those living, as Dr. Nicholls says, in "low-class industrial districts," but also those in better localities, who had big responsibilities, domestic or other, and who must, at least in many cases, have had to take up work which they heartily disliked rather than allow those near and dear to them to suffer want. As scientific psychologists we are perfectly aware that men's motives are highly complex, and to suggest that all panel men can be lumped together into one category, and all non-panel men into another, is nothing less than absurd; this sort of thing is worthy only of professional politicians, whose essential *rôle* in life is to persuade the gullible public to believe that black is white, out of "loyalty to party," or, as they commonly say, "patriotism."

It will be a sad day indeed for the country if the medical profession allows itself to drop to political levels. Dr. Malcolm Stewart well points out that the deterioration of the profession long antedated the Insurance Act. It takes two things to make a disease—soil and seed. A growing dislike of our art for its own sake, coupled with a growing appreciation of the emoluments to be derived from it, led to a soil being prepared upon which the political microbes, when they were ready, settled down with a *vim.* We are not going to cure this by forcing other people to be good. We are going to do it by throwing ourselves more keenly into our work, and concerning ourselves less with the methods by which our neighbours collar money that ought to be ours. In this way only we will regain that faith in ourselves which we so patently lack, and thus eventually we will secure the confidence of the public, which, despite all our talking, we do not hold at present.—I am, etc.,

ARTHUR J. BROCK, M.D.

Edinburgh, May 3, 1914.

